# Elevated temperature enhances task performance by improving cognitive abilities in common rudd (*Scardinius erythrophthalmus*)

**DOI:** 10.1038/s41598-025-92499-3

**Published:** 2025-03-05

**Authors:** Monika Sysiak, Ewa Babkiewicz, Marcin Lukasz Zebrowski, Katarzyna Rutkowska, Selvaraj Kunjiappan, Jae-Seong Lee, Piotr Maszczyk

**Affiliations:** 1 Department of Hydrobiology, Institute of Functional Biology and Ecology, Faculty of Biology, University of Warsaw, Żwirki i Wigury 101, 02-089 Warsaw, Poland; 2 Biological and Chemical Research Centre, University of Warsaw, Warsaw, Poland; 3 Department of Biotechnology, Kalasalingam Academy of Research and Education, Krishnankoil, 626126 India; 4 Department of Biological Sciences, College of Science, Sungkyunkwan University, Suwon, 16419 South Korea

**Keywords:** Ectotherms, Temperature, Task performance, Mobility, Cognitive abilities, Task difficulty, Ecology, Behavioural ecology, Climate-change ecology, Freshwater ecology

## Abstract

The thermal sensitivity of task performance in ectothermic organisms may depend on how temperature affects mobility, cognitive ability, or their interaction. Furthermore, these processes may vary with experience or task difficulty. To test these predictions, we performed mesocosm experiments with common rudd (*Scardinius erythrophthalmus*) foraging for a high-density food reward (*Artemia salina* nauplii) across consecutive daily sessions under varying task difficulties (short, medium, and long distances to the reward, and presence or absence of experienced individuals) at two temperatures (16–26 °C). Results indicated that the thermal sensitivity of task performance ranged from *Q*_*10*_ = 2 to 9 across all treatments, peaking during the second and third sessions when fish learned the reward location most intensively. *Q*_*10*_ values increased with task difficulty, reaching their highest levels when inexperienced fish navigated long distances to the reward and foraged without guidance. In contrast, the thermal sensitivity of mobility remained stable across sessions, with a maximum *Q*_*10*_ of 2. The significantly higher thermal sensitivity of task performance compared to mobility, along with its positive relationship with task difficulty, suggests that performance improvements at elevated temperatures are driven not only by increased mobility but also by enhanced cognitive processes.

## Introduction

A substantial body of literature explores the effects of various internal and external factors (both abiotic and biotic) on task performance across a wide range of animals, including numerous ectothermic species, primarily fish. These abilities support the integration of novel knowledge or behaviors, enabling individuals to navigate challenges, overcome obstacles, and achieve desired outcomes^1–3^. The impact of a given factor on task performance may depend on how it influences mobility, cognitive ability, or their interaction^1–3^. Typically, task performance begins with cognitive processes, which subsequently shape physical activity. For instance, under conditions of cannibalistic pressure, *Perca fluviatilis* learned to associate specific stimuli with threats and adjusted their behavior by becoming less exploratory^4^.

The mechanism by which temperature affects task performance of ectothermic organisms differs from that of other external factors, as it directly influences metabolic rates that subsequently influence both cognitive processes and mobility^5,6^. The relationship between temperature and metabolic rate can be represented by a hump-shaped curve, whereby the curve rises exponentially to a peak and then declines rapidly as the temperature moves from optimal to stressful^7–9^. It is widely accepted that the metabolic rate of an organism can be reliably extrapolated to other rates and processes at both individual and higher ecological levels^10–12^. The effect of temperature on biological processes in the ascending part of the thermal response curve can be expressed by the *Q*_*10*_ temperature coefficient, which represents the rate of increase of a given process per 10 °C increase in temperature^13^. A substantial body of research has demonstrated that the thermal sensitivity of a range of physiological and ecological processes is closely aligned with the *Q*_*10*_ value of 2^14,15^. This implies that for every 10 °C increase in temperature, the rate of biological processes doubles in an optimal temperature range. This elementary coefficient can be beneficial in comparing thermal sensitivities of different processes by assessing relative differences, and is widely employed in the thermal physiology literature^16^.

Given that the cognition and mobility of ectotherms are influenced by temperature-dependent metabolism^17,18^, it is reasonable to hypothesise that their thermal sensitivity, and thus the thermal sensitivity of task performance, would approximate *Q*_*10*_ = 2. However, cognition encompasses multiple sequential processes (perception, evaluation, learning, memorisation, and decision-making), and the thermal sensitivities of these processes may interact, potentially resulting in task performance that deviates from *Q*_*10*_ = 2. Furthermore, interactions between the thermal sensitivities of cognitive abilities and mobility could further influence task performance^6,19,20^. For instance, the swimming speed of fish increases with increasing temperature, driven not only by accelerated physiological processes but also by deliberate behavioural adjustments^19^. Moreover, faster-swimming fish at elevated temperatures encounter stimuli more frequently^20^.

Although numerous studies have examined how temperature affects task performance in endotherms, primarily humans^21,22^, relatively few have investigated this relationship in ectotherms, particularly within their optimal temperature range^23^. This is despite a substantial body of research on the thermal sensitivity of specific cognitive processes. For example, studies have shown that maternal exposure to elevated temperatures impairs the learning performance of juvenile rainbow trout (*Oncorhynchus mykiss*)^24^. Similarly, heat stress has been found to negatively affect the post-exposure learning performance of velvet gecko pups (*Amalosia lesueur*), and Australian skink (*Saiphos equalis*)^25^, as well as the memorisation abilities of fruit flies (*Drosophila melanogaster*)^26^. While the experimental design of some of the aforementioned studies considered task performance, it was primarily employed to investigate the thermal sensitivity of specific cognitive processes rather than task performance as an integrated outcome^20,27^. Moreover, the relative contributions of the thermal sensitivities of cognitive abilities and mobility to task performance, along with the interplay between these thermal sensitivities, remain poorly understood, particularly in terms of their influence on task performance over time. Assuming that the thermal sensitivity of cognitive processes influences task performance, it is likely that this effect is strongest at the outset of engaging with a novel task, before the solution has been automated. Another intriguing, yet unanswered question is whether the thermal sensitivity of task performance increases with task difficulty. Task difficulty can be influenced by factors such as the distance to a food reward^28^ or the presence or absence of experienced individuals, which reflect the influence of the social environment on task performance^29^. It can be expected that if cognitive processes are involved in task performance and cognitive abilities are positively affected by temperature, then the thermal sensitivity of task performance should increase with task difficulty. A partial answer to these questions can be provided by a simple behavioural experiment in which fish are acclimated to different temperatures and then their foraging for a food reward is recorded at different temperatures and task difficulty scenarios over several consecutive foraging sessions. Such an experiment enables the measurement of the thermal sensitivity of task performance, mobility, and learning rate in successive foraging sessions. By comparing the differences in rates of these processes and their variability over time, it is possible to infer changes in the influence of cognitive processes and mobility on the thermal sensitivity of task performance over time.

The aim of the study is to test three hypotheses concerning the relationship among the thermal sensitivity of task performance, cognitive abilities, and mobility in the planktivorous fish, common rudd (*S. erythrophthalmus*), under six different scenarios of varying task difficulty. These scenarios include three distinct distances to the food reward and the presence or absence of experienced individuals who are familiar with the reward’s location. We first hypothesize that fish will complete tasks faster at higher temperatures than at lower temperatures. We then expect that the positive effect of temperature on task performance is at least partially driven by improvements in cognitive processes. Finally, we expect that the thermal sensitivity of task performance will be more pronounced under more challenging conditions, specifically when the distance to the food reward is greater or when fish forage in the absence of experienced individuals.

## Results

### Swimming speed

**Fig. 1:**
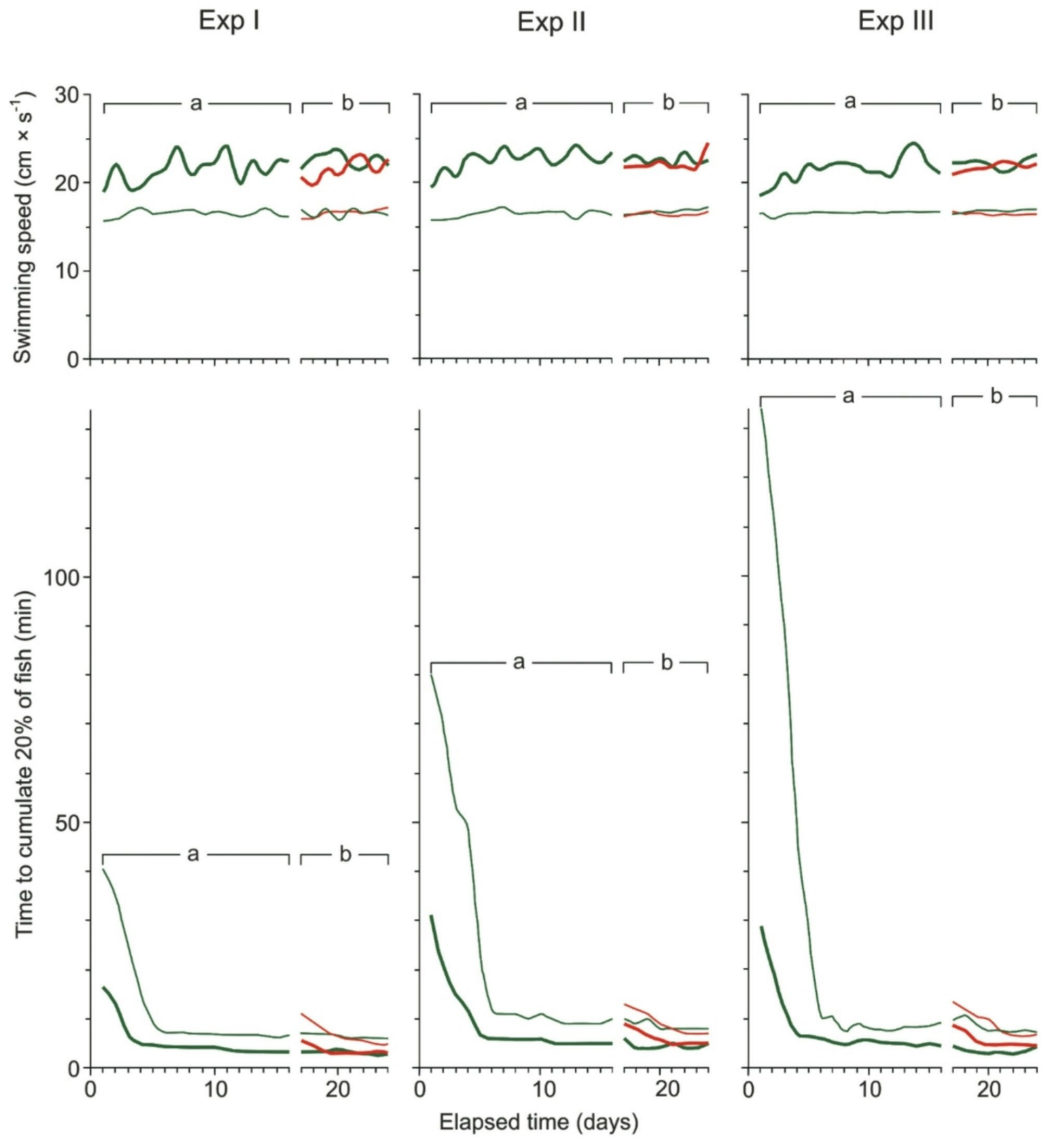
The time required to complete the task in the experiments with short (Exp I), medium (Exp II), and long (Exp III) distances between the start tank and the prey patch tank for 60 inexperienced fish (a, green lines) and 30 inexperienced fish (b, red lines) accompanied by 30 experienced fish at temperatures of 16 ºC (thin lines) and 26 ºC (thick lines).

Temperature and fish group type (i.e., inexperienced fish without and with an experienced fish) significantly affected swimming speed (GLMM, *p* < 0.001 for both factors, Table 1; Fig. 1). Furthermore, significant interactions were observed between temperature and fish group, as well as between temperature and distance to the patch (GLMM, *p* = 0 0.019 and 0.037 respectively, Table 1; Fig. 1). Specifically, swimming speed was higher at elevated temperatures than at lower temperatures for both groups of fish, across all distances to the patch (planned contrasts, *p* > 0.001, in all cases, Table 2; Fig. 1). Notably, the swimming speed of fish in groups with experienced individuals was significantly higher than that of fish in groups without experienced individuals, but only at higher temperatures for the short- and long-distance patch trials (planned contrast, *p* = 0.012 and 0.001, respectively, Table 2; Fig. 1). Furthermore, the distance to the patch also significantly influenced swimming speed; however, this effect was only observed at higher temperatures for fish groups without experienced individuals. Specifically, during the medium-distance patch trials, swimming speed was significantly greater than in the short- and long-distance trials (planned contrast, *p* = 0.025 and 0.013 respectively, Table 2; Fig. 1).

**Table 1:** Analysis of deviance from generalised linear mixed models with gamma distribution to test the effect of the temperature (T), fish group (with or without experienced individuals) (F), experiment with different distance to the patch (E) and their interactions on the fish swimming speed and time to accumulate 20% of fish in the high prey density tank (task performance).

Parameters	Factors; interactions	χ^2^	df	*p*
Swimming speed	T	**690.706**	**1.000**	**< 0.001**
	F	**26.459**	**1.000**	**< 0.001**
	E	4.959	2.000	0.084
	T × F	**4.341**	**1.000**	**0.037**
	T× E	**7.928**	**2.000**	**0.019**
	F × E	1.904	2.000	0.386
	T × F × E	2.616	2.000	0.270
Task performance	T	**65.799**	**1.000**	**< 0.001**
	F	**113.583**	**1.000**	**< 0.001**
	E	**25.063**	**2.000**	**< 0.001**
	T × F	**10.671**	**1.000**	**0.001**
	T × E	1.301	2.000	0.522
	F × E	2.254	2.000	0.324
	T × F × E	3.011	2.000	0.222

Statistically significant differences are shown in bold (χ^2^ - chi-squared statistic, df- degrees of freedom, *p* – *p*-value associated with a χ^2^).

### Task performance

Task performance, measured as the time required to accumulate 20% of the fish in the high zooplankton density tank, was significantly influenced by temperature, fish group type, and distance to the patch (GLMM, *p* < 0.001 for each factor, Table 1; Fig. 1). However, the only significant interaction observed was between temperature and fish group type (GLMM, *p* = 0.001, Table 1; Fig. 1). Accumulation time was significantly shorter at higher temperatures than at lower temperatures, but this effect was limited to the group of inexperienced fish (without experienced individuals) and was consistent across all distances to the patch (planned contrast, *p* = 0.007, 0.001 and < 0.001 for short, medium and long distances respectively, Table 2; Fig. 1). At lower temperatures, fish groups with experienced individuals exhibited significantly shorter accumulation times compared to those without experienced individuals, across all distances to the patch (planned contrast, *p* < 0.001, Table 2, for each comparison). Similarly, at higher temperatures, the accumulation time was also significantly shorter for groups with experienced individuals, but only for the short- and medium-distance patches (*p* = 0.034 and 0.027, respectively, Table 2; Fig. 1).

**Table 2:** Planned contrasts for estimated marginal means using generalised linear mixed models with gamma distribution to assess the differences in swimming speed and time to accumulate 20% of fish in the high prey density tank (task performance) between treatments, which are combinations of the three variables: the temperature (16 and 26 °C), fish group (F1 – inexperienced fish without experienced individuals, F2 – inexperienced fish with experienced individuals) and the experiment with different distance to the patch (E1 – short distance, E2 – medium distance, E3 – long distance).

Parameter	Contrasts	E	SE	T	*p*
Swimming speed	T16×F1×E1 –T26×F1×E1	**-0.197**	**0.024**	**-8.326**	**< 0.001**
	T16×F2×E1 –T26×F2×E1	**-0.269**	**0.024**	**-11.386**	**< 0.001**
	T16×F1×E2 –T26×F1×E2	**-0.293**	**0.024**	**-12.397**	**< 0.001**
	T16×F2×E2 –T26×F2×E2	**-0.291**	**0.024**	**-12.310**	**< 0.001**
	T16×F1×E3 –T26×F1×E3	**-0.211**	**0.024**	**-8.913**	**< 0.001**
	T16×F2×E3 –T26×F2×E3	**-0.261**	**0.024**	**-11.044**	**< 0.001**
	T16×F1×E1 – T16×F2×E1	-0.008	0.024	-0.320	1.000
	T16×F1×E2 – T16×F2×E2	-0.038	0.024	-1.614	1.000
	T16×F1×E3 – T16×F2×E3	-0.043	0.024	-1.815	0.974
	T26×F1×E1 – T26×F2×E1	**-0.080**	**0.024**	**-3.380**	**0.012**
	T26×F1×E2 – T26×F2×E2	-0.036	0.024	-1.526	1.000
	T26×F1×E3 – T26×F2×E3	**-0.093**	**0.024**	**-3.945**	**0.001**
	T16×F1×E1 – T16×F1×E2	0.022	0.024	0.922	1.000
	T16×F1×E1 – T16×F1×E3	0.019	0.024	0.790	1.000
	T16×F1×E2 – T16×F1×E3	-0.003	0.024	-0.132	1.000
	T26×F1×E1 – T26×F1×E2	**-0.074**	**0.024**	**-3.149**	**0.025**
	T26×F1×E1 – T26×F1×E3	0.005	0.024	0.203	1.000
	T26×F1×E2 – T26×F1×E3	**0.079**	**0.024**	**3.352**	**0.013**
	T16×F2×E1 – T16×F2×E2	-0.009	0.024	-0.372	1.000
	T16×F2×E1 – T16×F2×E3	-0.017	0.024	-0.705	1.000
	T16×F2×E2 – T16×F2×E3	-0.008	0.024	-0.333	1.000
	T26×F2×E1 – T26×F2×E2	-0.031	0.024	-1.295	1.000
	T26×F2×E1 – T26×F2×E3	-0.009	0.024	-0.362	1.000
	T26×F2×E2 – T26×F2×E3	0.022	0.024	0.933	1.000
Task performance	T16×F1×E1 –T26×F1×E1	**0.964**	**0.271**	**3.556**	**0.007**
	T16×F2×E1 –T26×F2×E1	0.706	0.271	2.604	0.120
	T16×F1×E2 –T26×F1×E2	**1.141**	**0.271**	**4.208**	**0.001**
	T16×F2×E2 –T26×F2×E2	0.428	0.271	1.579	0.801
	T16×F1×E3 –T26×F1×E3	**1.674**	**0.271**	**6.172**	**< 0.001**
	T16×F2×E3 –T26×F2×E3	0.475	0.271	1.751	0.720
	T16×F1×E1 – T16×F2×E1	**1.091**	**0.271**	**4.024**	**< 0.001**
	T16×F1×E2 – T16×F2×E2	**1.569**	**0.271**	**5.784**	**< 0.001**
	T16×F1×E3 – T16×F2×E3	**1.965**	**0.271**	**7.246**	**< 0.001**
	T26×F1×E1 – T26×F2×E1	**0.833**	**0.271**	**3.072**	**0.034**
	T26×F1×E2 – T26×F2×E2	**0.856**	**0.271**	**3.155**	**0.027**
	T26×F1×E3 – T26×F2×E3	0.766	0.271	2.825	0.066
	T16×F1×E1 – T16×F1×E2	-0.774	0.271	-2.855	0.065
	T16×F1×E1 – T16×F1×E3	**-1.168**	**0.271**	**-4.307**	**< 0.001**
	T16×F1×E2 – T16×F1×E3	-0.394	0.271	-1.452	0.879
	T26×F1×E1 – T26×F1×E2	-0.598	0.271	-2.203	0.331
	T26×F1×E1 – T26×F1×E3	-0.459	0.271	-1.691	0.727
	T26×F1×E2 – T26×F1×E3	0.139	0.271	0.512	1.000
	T16×F2×E1 – T16×F2×E2	-0.297	0.271	-1.095	1.000
	T16×F2×E1 – T16×F2×E3	-0.294	0.271	-1.085	1.000
	T16×F2×E2 – T16×F2×E3	0.003	0.271	0.010	1.000
	T26×F2×E1 – T26×F2×E2	-0.575	0.271	-2.120	0.374
	T26×F2×E1 – T26×F2×E3	-0.526	0.271	-1.938	0.526
	T26×F2×E2 – T26×F2×E3	0.049	0.271	0.182	1.000

Statistically significant differences are shown in bold (E – estimate, SE – standard error, Z –statistic, p – *p*-value).

Accumulation time in the high prey density tank was also significantly influenced by the distance to the patch. Specifically, accumulation time was significantly shorter in the short-distance trial compared to the long-distance trial, but this effect was observed only at the lower temperature for the group of fish without experienced individuals (planned contrast, *p* < 0.001, Table 2; Fig. 1).

### *Q*_*10*_ value for the swimming speed

**Fig. 2:**
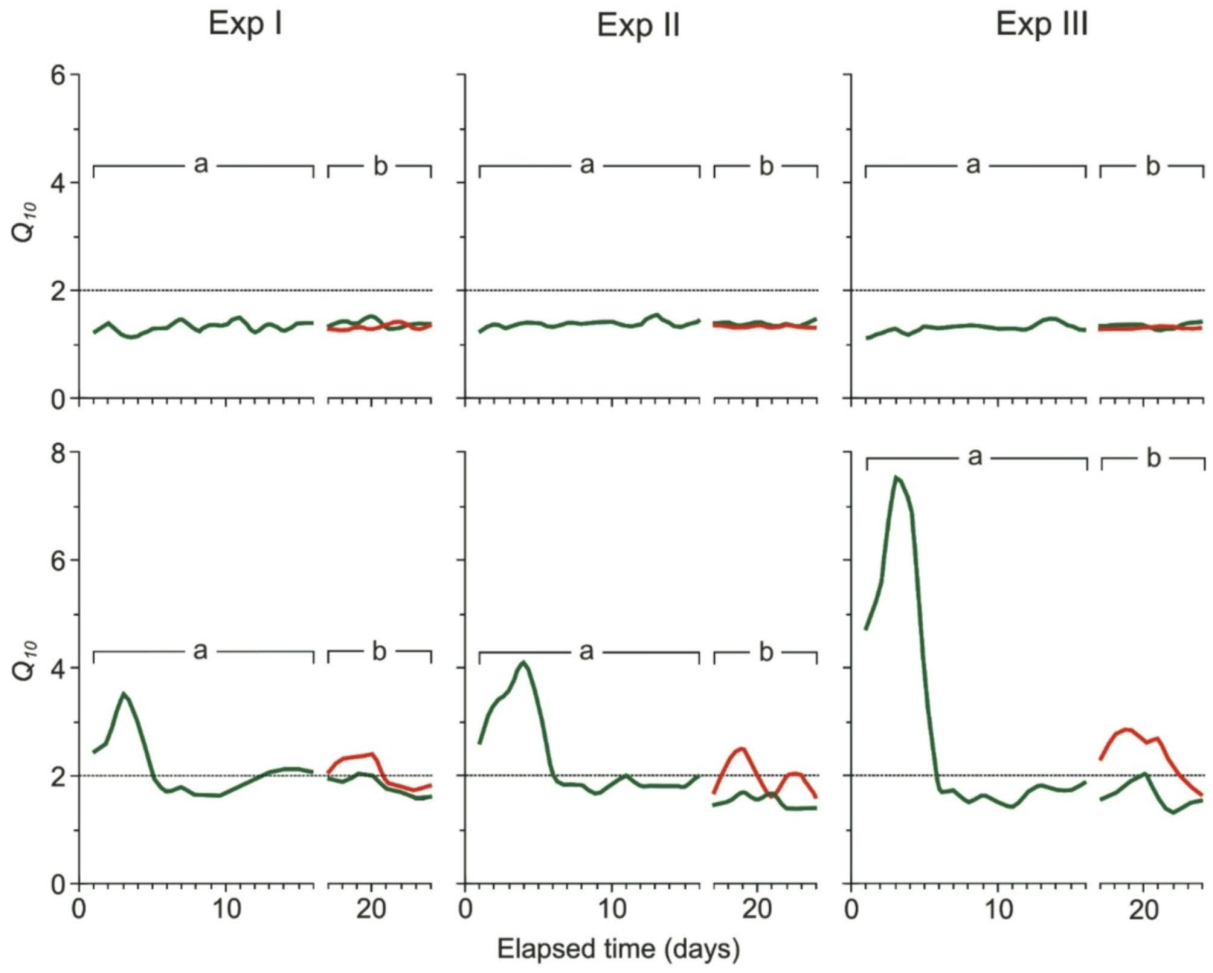
The *Q*_*10*_ values for the time required to complete the task in the experiments with short (Exp I), medium (Exp II), and long (Exp III) distances between the start tank and the prey patch tank for 60 inexperienced fish (a, green lines) and 30 inexperienced fish (b, red lines) accompanied by 30 experienced fish at temperatures of 16 ºC (thin lines) and 26 ºC (thick lines).

Fish group type and distance to the patch had a significant effect on *Q*_*10*_ value (GLMM, *p* = 0.004 and < 0.001 respectively, Table 3; Fig. 2), with no significant interaction between these factors. Specifically, the Q10 value for swimming speed was significantly higher in the group of fish with experienced individuals compared to the group without experienced individuals, but this difference was only evident in the experiment involving the shortest distance to the patch (planned contrast, *p* = 0.021, Table 4; Fig. 2). Additionally, in the group of fish without experienced individuals, the Q10 value was significantly higher in the experiment with the medium distance to the patch compared to those with short and long distances (planned contrast, *p* = 0.001 and 0.005 respectively, Table 4; Fig. 2). In contrast, no significant differences in the Q10 value were observed across experiments with varying distances to the patch for the group of fish with experienced individuals (planned contrast, Table 4; Fig. 2).

**Table 3:** Analysis of deviance from generalised linear mixed models with gamma distribution to test the effect of the fish group (F), distance to the patch (E) and their interaction on the *Q*_*10*_ coefficients for: fish swimming speed (SS), time to accumulate 20% of fish in the high prey density tank (task performance, TP) and the ratio of these two measures.

Parameters	Factors; interaction	χ^2^	df	*p*
*Q*_*10*_ for the SS	F	**8.466**	**1.000**	**0.004**
	E	**15.276**	**2.000**	**< 0.001**
	F × E	4.881	2.000	0.087
*Q*_*10*_ for the TP	F	**82.546**	**1.000**	**< 0.001**
	E	**9.166**	**2.000**	**0.010**
	F × E	**22.603**	**2.000**	**< 0.001**
*Q*_*10*_ for the ratio between TP and SS	F	**86.975**	**1.000**	**< 0.001**
	E	**11.516**	**2.000**	**0.003**
	F × E	**20.999**	**2.000**	**< 0.001**

Statistically significant differences are shown in bold (χ^2^ - chi-squared statistic, df- degrees of freedom, *p* – *p*-value associated with a χ^2^).

**Table 4:** Planned contrasts for estimated marginal means using generalised linear mixed models with gamma distribution to assess the differences in *Q*_*10*_ coefficients for fish swimming speed (SS), time to accumulate 20% of fish in the high-prey density tank – task performance (TP) and the ratio of these two measures, which are combinations of the two variables: the fish group (F1 – inexperienced fish without experienced individuals, F2 – inexperienced fish with experienced individuals) and experiments with different distance to the patch (E1 – short distance, E2 – medium distance, E3 – long distance).

Parameter	Contrast	E	SE	T	*p*
*Q*_*10*_ for the SS	F1×E1 – F2×E1	**-0.071**	**0.024**	**-2.965**	**0.021**
	F1×E2 – F2×E2	0.001	0.024	0.059	1.000
	F1×E3 – F2×E3	-0.051	0.024	-2.134	0.197
	F1×E1 – F1×E2	**-0.094**	**0.024**	**-3.948**	**0.001**
	F1×E1 – F1×E3	-0.012	0.024	-0.504	1.000
	F1×E2 – F1×E3	**0.082**	**0.024**	**3.444**	**0.005**
	F2×E1 – F2×E2	-0.022	0.024	-0.924	1.000
	F2×E1 – F2×E3	0.008	0.024	0.327	1.000
	F2×E2 – F2×E3	0.030	0.024	1.252	1.000
*Q*_*10*_ for the TP	F1×E1 – F2×E1	0.251	0.135	1.856	0.254
	F1×E2 – F2×E2	**0.717**	**0.135**	**5.303**	**< 0.001**
	F1×E3 – F2×E3	**1.160**	**0.135**	**8.578**	**< 0.001**
	F1×E1 – F1×E2	-0.197	0.135	-1.457	0.294
	F1×E1 – F1×E3	**-0.685**	**0.135**	**-5.068**	**< 0.001**
	F1×E2 – F1×E3	**-0.488**	**0.135**	**-3.611**	**0.002**
	F2×E1 – F2×E2	0.269	0.135	1.990	0.233
	F2×E1 – F2×E3	0.224	0.135	1.655	0.294
	F2×E2 – F2×E3	-0.045	0.135	-0.335	0.738
*Q*_*10*_ for the ratio between TP and SS	F1×E1 – F2×E1	0.316	0.139	2.265	0.117
	F1×E2 – F2×E2	**0.719**	**0.139**	**5.154**	**< 0.001**
	F1×E3 – F2×E3	**1.218**	**0.139**	**8.734**	**< 0.001**
	F1×E1 – F1×E2	-0.110	0.139	-0.787	0.862
	F1×E1 – F1×E3	**-0.684**	**0.139**	**-4.904**	**< 0.001**
	F1×E2 – F1×E3	**-0.574**	**0.139**	**-4.117**	**< 0.001**
	F2×E1 – F2×E2	0.293	0.139	2.101	0.143
	F2×E1 – F2×E3	0.218	0.139	1.564	0.354
	F2×E2 – F2×E3	-0.075	0.139	-0.537	0.862

Statistically significant differences are shown in bold (E – estimate, SE – standard error, Z –statistic, *p* – *p*-value).

### ***Q***_***10***_**value for the task performance**

The *Q*_*10*_ value for the accumulation time differed significantly between the fish groups with and without experienced individuals, as well as between the experiments with different distances to the patch (GLMM, *p* < 0.001 and 0.010 respectively, Table 3; Fig. 2). A significant interaction effect between these two factors was also observed (GLMM, *p* < 0.001, Table 3; Fig. 2). Specifically, the *Q*_*10*_ value for accumulation time was significantly higher for the group of fish without experienced individuals compared to the group with experienced individuals in the medium and long-distance patch experiments (planned contrast, *p* < 0.001 for both comparisons, Table 4; Fig. 2). The *Q*_*10*_ value related to the distance to the patch differed significantly only for the group of fish without experienced individuals, being higher in the long-distance experiment compared to both the short and medium distances (planned contrast, *p* < 0.001 and 0.002, Table 4; Fig. 2).

### *Q*_*10*_ value for the ratio between task performance and swimming speed

Fish group and distance to the patch significantly affected the *Q*_*10*_ ratio (GLMM, *p* < 0.001 and 0.003, Table 3; Fig. 2), with an interaction between these two factors also observed (GLMM, *p* < 0.001, Table 3; Fig. 2). The *Q*_*10*_ ratio was significantly higher for the group of fish without experienced individuals compared to the group of fish with experienced individuals for both the medium and long-distance patch experiments (planned contrast, *p* < 0.001 for both comparisons, Table 4; Fig. 2). The *Q*_*10*_ coefficient differed significantly across experiments with varying distances to the patch only for the group of fish without experienced individuals, being greater in the long-distance experiment compared to both the short and medium distance experiments (planned contrast, *p* < 0.001, Table 4; Fig. 2).

### *Q*_*10*_ value for the daily rate of changes in task performance

The *Q*_*10*_ value of the daily rate of changes in task performance for the first session exhibited a range of 1.47 to 2.58 for the fish group without the experienced individuals while the range with experienced individuals was 1.21 to 1.44 (Table 5). The descriptive statistics revealed that the mean value of the *Q*_*10*_ for the first three consecutive foraging sessions exceeded 1.0 for each of the fish groups and distances to the patch, indicating a positive impact of temperature on task performance. The mean values for the fish group without experienced individuals were 1.27 ± 0.51, 2.14 ± 0.83 and 1.48 ± 0.50, while the mean values for the fish group with experienced individuals were 1.00 ± 0.42, 1.43 ± 0.83 and 2.10 ± 1.22, respectively, for the short, medium and long distances to the patch (Table 5). However, throughout all seven sessions (the complete testing period for each fish group), the *Q*_*10*_ value for the daily rate of change was close to or even below zero. For the group of fish without experienced individuals, the mean *Q*_*10*_ values were 0.73 ± 0.75, 1.10 ± 0.97 and 0.86 ± 0.82 for the short, medium, and long distances to the patch, respectively. The group of fish with experienced individuals exhibited mean values of 0.97 ± 0.51, 0.80 ± 0.83 and 0.85 ± 1.68 for the corresponding distances (Table 5).

**Table 5:** The descriptive statistic for the *Q*_*10*_ coefficient for the daily rate of change in task performance for the first foraging session, the first three of the foraging sessions and the first seven of the foraging sessions for the fish group (fish without experienced individuals and with experienced individuals) and different distances to the patch (Exp I - short, exp II - medium and exp III - long).

Fish group	Distance to the patch	First session	1–3 sessions		1–7 sessions	
			Mean	SD	Mean	SD
Without experienced individuals	Short	1.47	1.27	0.51	0.73	0.75
	Medium	2.58	2.14	0.83	1.10	0.97
	Long	1.74	1.48	0.50	0.86	0.82
With experienced individuals	Short	1.33	1.00	0.42	0.97	0.51
	Medium	1.44	1.43	0.83	0.80	0.83
	Long	1.21	2.10	1.22	0.85	1.68

## Discussion

The findings of the study supported the first hypothesis, as the completion of the task was observed to occur at a slower rate at low temperature in comparison to high temperature, even during the final foraging sessions when the fish had demonstrated the ability to locate the food reward at the maximum possible rate. The positive effect of temperature on task performance is consistent with the reanalysis of data from our previous studies on *D. rerio* in the temperature range from 21 to 31 °C^20^, and on common rudd in the temperature range from 16 to 26 °C^19^. However, a reanalysis of data from other studies demonstrated a negative or insignificant effect of temperature on task performance in ectothermic fish species (*Gambusia affinis*)^23^. This discrepancy is likely attributable to the fact that our studies employed a temperature range that did not exceed the thermal optimum for the fish, whereas the majority of previous studies tested the effects of suboptimal temperatures^30^.

The thermal sensitivity of task performance notably exceeded *Q*_*10*_ = 7, suggesting that this phenomenon is not solely driven by temperature-dependent physiological processes. Given that our experiments involved groups of fish, the high thermal sensitivity of task performance might initially appear to stem from the pronounced thermal sensitivity of interactions within the group^19,20^. However, in the light of a previous study^20^, the conclusion is the opposite. Although the previous study did not include an analysis of the thermal sensitivity of task performance, a re-analysis of the results for this parameter for *D. rerio* foraging individually revealed even higher values than in the present study. These values ranged from 19.9 to 12.4 for different measured parameters. The analysis of the course of thermal sensitivity of task performance in subsequent foraging sessions demonstrated that the observed effect is not solely a result of temperature-dependent physiological processes, as the value was much greater than the value of *Q*_*10*_ = 2. In the initial session, the sensitivity was approximately three to four times higher at the elevated temperature, and it increased further between sessions two and four. Subsequently, a notable decline in sensitivity was observed, reaching a level that was twice as high as that observed at the lower temperature in the remaining sessions. This pattern may indicate changes in the thermal sensitivity of the learning rate during successive foraging sessions, consistent with the anticipated role of learning, which is likely most pronounced after the fish have encountered the food reward but before they have achieved maximum efficiency in locating it. These findings confirm our predictions outlined in the second hypothesis.

An additional rationale for the positive impact of learning on task performance at elevated temperatures can be derived from an examination of the discrepancy between the influence of temperature on task performance and mobility. The findings indicated that the thermal sensitivity of the fish’s swimming speed, a measure of mobility, exhibited a range of *Q*_*10*_ between 1.4 and 1.6, with the highest values observed in the latest foraging sessions. These findings are consistent with those of previous studies, which demonstrated that the thermal sensitivity of swimming speed in *D. rerio* ranged from 1.6 to 2.3 between 21 and 31 °C^20^ and in common rudd, it ranged from 1.0 to 1.5 between 16 and 26 °C^19^. Thus, it can be concluded that the discrepancy in thermal sensitivity between task performance and mobility (i.e., a high *Q*_*10*_ ratio for task performance compared to mobility) provides compelling evidence that the enhanced task performance at elevated temperatures is not solely attributable to an increase in fish mobility, but also to an augmentation in learning rate, either independently^31^ or in combination with increased mobility^32^. The interaction may be explained by the fact that an elevated metabolic rate would result in enhanced mobility, which in turn would facilitate more frequent and more rapid stimulus encounters, thereby enhancing cognitive performance to a greater extent than would result from temperature-dependent physiological processes alone^5,6^.

Additionally, the findings indicated that the thermal sensitivity of the daily rate of change in task performance fell within the range of *Q*_*10*_ = 1.0 and 2.6 during the initial foraging session and within the range of *Q*_*10*_ = 1.0 and 2.1 during the first three sessions in each experiment for both groups of fish (foraging in the presence or absence of the experienced fish). This change may be attributed to an increased metabolic rate^33,34^, which could consequently result in increased hunger and motivation to forage^35^ in fish exposed to elevated temperature (26 ℃). However, this hypothesis appears implausible in the light of the fact that the fish were provided with a standard and relatively high amount of food between foraging sessions. A more plausible explanation for the observed temperature effect on the daily rate of change in successive foraging sessions is that the elevated temperature increased the spatial learning rate of the fish, including their ability to learn the location of food patches and to habituate to the experimental system. These findings are consistent with those of our previous study, which demonstrated that the thermal sensitivity of the learning rate in *D. rerio* between 21 and 31 °C ranged from *Q*_*10*_ = 1.6 to 2.3 for different parameters representing the spatial learning rate^20^. It is notable that the thermal sensitivity of learning rates in the final four foraging sessions of our study was below *Q*_*10*_ = 1.0, indicating that fish exhibited accelerated learning at low temperature (and slower in elevated temperature). This implies that fish at high temperature achieved a more expeditious level of proficiency in locating food rewards, although both temperatures ultimately resulted in a comparable level of proficiency.

The most likely explanation for the positive effect of temperature on the learning rate of the fish during the initial foraging sessions observed in the study is based on the specificity of the spatial learning tests. With each subsequent foraging session, the fish subjected to the high temperature treatment exhibited a reduction in foraging time and an earlier encounter with the food reward. As a result, they were presented with a more extensive opportunity to learn about the stimuli by the conclusion of the foraging session. This may have contributed to the more efficient memorisation of the location of the food reward. This interpretation is supported by the findings of Angiulli et al. (2020)^36^, which demonstrated that elevated temperatures diminish anxiety and enhance boldness in zebrafish, enabling them to remain in potentially perilous areas of the tank for extended periods.

The increasing thermal sensitivity of task performance with increasing difficulty is consistent with the predictions of our third hypothesis. Since the thermal sensitivity of mobility was similar across varying distances to the food reward, the observed increase in thermal sensitivity with greater distance can be attributed to a stronger reliance on learning rate. Moreover, the presence of experienced fish significantly accelerated task performance, particularly at lower temperatures, suggesting that fish benefit more from the presence of experienced individuals in colder environments. Interestingly, the positive effect of temperature on task performance was much more pronounced for inexperienced fish without experienced companions (i.e., in more challenging scenarios). Given that the thermal sensitivity of mobility was similar for inexperienced fish, whether they had companions or not, the higher thermal sensitivity of task performance in fish without experienced companions can be attributed to a greater reliance on learning and memory. This is likely because inexperienced fish, when without companions, are forced to explore and make decisions independently, which actively engages them in learning and memory processes, and these processes become more evident at elevated temperatures.

In conclusion, the results demonstrated that at temperatures not exceeding the optimum, fish solved tasks more quickly at higher temperatures, which was due not only to their increased mobility but also to enhanced cognitive abilities, including learning rate. Based on our study, it can thus be concluded that elevated temperatures have a greater impact on task performance in ectothermic organisms that are more mobile and possess greater cognitive abilities. Furthermore, our results also suggest that the thermal sensitivity of task performance depends more on how the learning rate changes with increasing temperature than on mobility. The differences in temperature effects may have significant ecological consequences at various levels. For example, at the population level, elevated temperatures may promote individuals with greater cognitive abilities, despite their lower mobility compared to other individuals. At the community level, the increased efficiency in task solving at elevated temperatures may explain the well-established observation in the literature that the foraging efficiency of planktivorous fish increases with rising temperatures^37–40^.

## Materials and methods

### Experimental animals

Three behavioural experiments were conducted using juvenile common rudd (*S. erythrophthalmus*) as the model organism. A substantial body of prior research has been conducted on this species, examining its physiological and behavioural responses to temperature changes^19,41^. A total of 180 individuals were utilised in each experiment. Prior to the commencement of the experiments, the fish were weighed while anaesthetised with buffered MS-222 (tricaine methanesulfonate) at a concentration of 100 mg × L^− 1^. The mean fresh weight of the fish was 2.01 ± 0.42 g (Exp I), 1.82 ± 0.30 g (Exp II) and 2.32 ± 0.68 g (Exp III).

The red and yellow elastomer tags (Visible Implant Elastomer (VIE) Tags, Northwest Marine Technology, Inc., USA) were prepared in accordance with the manufacturer’s recommended procedure. Subsequently, the tags were injected into the anaesthetised fish as a thin strip just under the skin using insulin syringes, in close proximity to the dorsal fin on both sides. This enabled the differentiation between groups of experienced and naive fish when they were combined in an experimental system. The fish that had not been tagged with the elastomer were administered a physiological salt injection (0.06% sodium chloride dissolved in distilled water) in the same body part. The unmarked fish were employed in order to facilitate the replacement of half the fish in the second stage of the experiment with naive fish, while maintaining the same overall number of fish in both stages of the experiment. The fish marked with yellow elastomer served as naive fish during the first stage of the experiment and as experienced fish during the subsequent stage. Conversely, the fish marked with red elastomer performed the function of naive fish during the latter stage of the experiment. The selected unmarked and marked fish were acclimated to a specified temperature for a minimum of seven days. Subsequently, the fish were provided with a limited quantity of frozen *Chironomidae* sp. larvae and *Artemia salina* nauplii, administered in equal proportions across the entire system. The prey utilized during the foraging sessions was *A. salina* nauplii, hatched daily from dried brine shrimp cysts (Sanders Brine Shrimp Company, Inc.), sourced from the fishless Great Salt Lake in Utah, USA. The two-day-old nauplii remained viable and motile in the freshwater system for a period of 24 h. The established experimental temperatures of 16 and 26 °C were selected to encompass the optimal temperature range for this species, with the lower temperature falling within this range and the higher one slightly above it. This approach is supported by previous research^42–44^.

### Experimental system

The experimental system consisted of two identical sections. Each section was constituted of ten tanks, with each tank containing 200 L of tap water. The tanks were rectangular in shape and connected to one another via circular openings with a diameter of 8 cm. The openings were furnished with rotatory blinds, thereby enabling the openings to be opened and closed at any desired point in time. The system maintained: (1) a uniform light intensity of 0.8 ± 0.2 µmol × m⁻² × s⁻¹ at a depth of 0.5 m, and (2) a constant water temperature, with one section at 16 °C and another at 26 °C. The movements of fish within the final tank, which contained a food reward, were recorded using high-resolution infrared cameras (P.P.H. Matar KT-370/540, Poland) connected to a computer and positioned 0.5 cm below the water surface. A detailed description of the system can be found in our previous study^19^. The number of tanks employed in the experiments differed, with four tanks used in Experiment 1, six in Experiment II, and eight in Experiment III. This resulted in a variation in the distance between the initial tank and the final tank, which contained the food reward (a high density of *A. salina* nauplii). The distance was 1.3 m in Experiment I (three obstacles to overcome), 1.6 m in Experiment II (five obstacles to overcome) and 2.8 m in Experiment III (seven obstacles to overcome). During the experiments, the outer blinds were closed in the start and last tank, thereby enabling the fish to leave the tanks only in one direction.

### Experimental procedure

The experiments were conducted in two distinct phases. In the initial phase of the experiment, 30 fish marked with yellow elastomer and 30 unmarked fish were placed in each section and permitted to learn to locate the food patch over the course of 16 daily foraging sessions, with the temperature set at either 16–26 °C. In the second stage of the experiment, 30 marked fish with red elastomer were randomly substituted for the 30 unmarked fish. Over the following eight days, the recently introduced fish were permitted to follow the cues in the presence of 30 individuals with prior experience.

Seven days prior to the commencement of the experiments, the fish were introduced to the starter tank, with one group allocated to the section with a low temperature and the second group to the section with a high temperature, in order to facilitate their acclimatisation to the experimental temperature. Prior to the commencement of each experiment, the water flow through the tanks was temporarily halted. The fish that had been labelled with elastomer for utilisation in the second stage of the experiment were captured and transferred to one of the tanks that had not been utilised during the experiment.

The same experimental procedure employed by Gliwicz and Maszczyk (2016)^19^ was utilised to provide the fish with patchily distributed *Artemia* prey. Just before the start of each foraging session, 91% of the total number of *Artemia* was introduced into the high-density tank, while 1% of the total number of *Artemia* was added to each of the other tanks. The *Artemia* in the other tanks were introduced to encourage the fish to forage and search for a food patch. The experiment commenced with the opening of the connection between the starting tank and the subsequent experimental tank. The timer was activated, and camera recording began, allowing the fish to occupy the experimental tank unaccompanied.

Each foraging session lasted 60 min. The fish in the tank with the food patch were recorded at one-minute intervals throughout each foraging session to determine the time taken to complete the task and to measure the swimming speed of the fish. At the conclusion of each session, video recording was stopped, and water flow through the tanks was restored. The fish were then transferred back to the starting tank, and the connection between the starting tank and the adjacent tank was closed.

The experimental procedure was approved by the First Warsaw Local Ethical Committee for Animal Experiments (permission protocol no. 042/2016). All methods were performed in accordance with the relevant guidelines and regulations. The study is reported in accordance with ARRIVE guidelines.

### Data analysis

The time to complete the task and the swimming speed of the fish (m × s⁻¹) were determined by analyzing archived video footage. It was determined that the task was completed during each foraging session when 20% of the fish marked in yellow during the first phase of the experiment and 20% of the fish marked in red during the second phase of the experiment were present in the tank with a food patch. The 20% threshold was adopted based on the experimental procedure used in our previous study^19^. This threshold was sufficient to reliably estimating the accumulation rate of the fish, while also ensuring that the foraging session ended before there was a significant reduction in food (*Artemia*) density in the high-density tank.

The fish swimming speed was measured by comparing two successive images, with the distance traversed by an individual fish in the lower 20 cm of the tank quantified using a scale marked at the tank’s base. The distance was then divided by the elapsed time to obtain the swimming speed.

The impact of temperature on fish performance, encompassing both task performance and speed, was quantified as a temperature coefficient (*Q*_10_), calculated in accordance with the formula proposed by Schmidt-Nielsen (1979)^14^. The temperature coefficient (*Q*_10_) was calculated using the following formula: *Q*_10_ = (*R2/R1*)^(*10/T2 − T1*)^, where *R2* is the measured reaction rate at temperature *T2* (where *T2* > *T1*) and *R1* is the measured reaction rate at temperature *T1*.

The statistical analysis was conducted using the R software, version 4.3.2 (R Core Team, 2023). A 5% statistical significance level was set. The data set under analysis encompasses the initial six days following the introduction of the fish into the experimental system. The effects of temperature, fish group, distance to the patch, and their interactions on task performance were analyzed using generalized linear models (GLM)^45^, performed with the Template Model Builder (the glmmTMB package v.1.1.3)^46^. Temperatures (16 and 26 °C), fish group (fish without and with experienced individuals), and distance to the patch (short, medium, and long distances) were set as fixed effects. Task performance, measured as the time required to accumulate 20% of the fish in the high prey density tank, was designated as the response variable.

The same type of model was also used to test the significance of fixed effects: fish group (fish without and with experienced individuals) and distance to the patch (short, medium and long distances) on the *Q*_*10*_ coefficients for fish swimming speed, time to accumulate 20% of fish in the high prey density tank, and the ratio of these two measures.

All count data were modeled with a gamma distribution with log link function. The models fitting to the dataset were diagnosed using DHARMa scaled residual plots (the *DHARMa* package v.0.4.5)^47^. Significance of interactions between the factors was assessed using analysis of deviance and the Wald type II chi-squared difference test (χ2) (the *car* package v.3.0–12)^48^. *Post-hoc* multiple comparisons were based on the planned contrasts for estimated marginal means (EMMs; the *emmeans* package (v.1.7.2.)^49^. The Holm *p*-value adjustment was applied to control for type I error inflation due to multiple testing.

The *Q*_*10*_ for the daily rate of change in task performance during the first foraging session, the first three of the foraging sessions, and the first seven foraging sessions, for each fish group (fish with and without experienced individuals) and across different distances to the patch (short, medium, and long), were presented using descriptive statistics.

## Data Availability

The data will be made available upon request. All requests for data from this study should be directed to P.M.
